# The phosphatase of regenerating liver-3 protein (PRL-3) promotes glioma cell invasiveness by interacting with β3 –tubulin

**DOI:** 10.1080/21655979.2021.2001220

**Published:** 2022-02-12

**Authors:** Zhenying Ge, Tingxuan Gu, Lingge Zhang, Qingfang Fan, Li Ma, Na Fang

**Affiliations:** a Laboratory of Cell Signal Transduction, Henan Provincial Engineering Centre for Tumor Molecular Medicine, Medical School of Henan University, Kaifeng, China; b Kaifeng Key Laboratory of Cell Signal Transduction, Kaifeng Science & Technology Bureau, Kaifeng, China; c Department of Pathophysiology, School of Basic Medical Sciences, Zhengzhou University, Zhengzhou, China; d Department of Pathophysiology, China-US (Henan) Hormel Cancer Institute, Zhengzhou, China; e Department of Hematology, Xinxiang Central Hospital, Xinxiang, China; f Department of Infectious Diseases, Henan Provincial People’s Hospital, Henan University, Zhengzhou, China

**Keywords:** PRL-3, glioma, β3-tubulin, dephosphorylation, migration

## Abstract

PRL-3 is a tyrosine phosphatase linked with tumor metastasis. It is detected high expression in different kinds of cancers, including colorectal, gastric, ovarian, and liver cancer. Its high expression is positively correlated with the progression of tumors and negatively with survivals of patients. However, the detailed mechanism underlying PRL-3 in tumor metastasis still remains unclear. In the present study, we found that PRL-3 is able to bind to β3-tubulin in pull-down and co-immunoprecipitation assays. Furthermore, overexpression of PRL-3 dephosphorylated β3-tubulin, a component of cytoskeleton, which plays critical role in cell shape formation and migration. Using cell wound healing and matrigel invasion assays, we found that PRL-3 could promote the migration and invasion of glioma cells. Taken together, our study revealed that PRL-3 may be involved in migration and invasion of glioma by dephosphorylating β3-tubulin. It is tempting to speculate that dephosphorylation of β3-tubulin by PRL-3 results in assembly of the cytoskeleton and facilitates cell migration and/or tumor metastasis.

## Introduction

Glioma is one of the most common brain tumors in adults and is characterized by high aggressiveness and poor prognosis. The standard treatment of glioma remains unsatisfactory, due to its infiltration into surrounding normal brain tissue and high risk of recurrence after treatments. In addition to surgery, radiotherapy, and chemotherapy, recent studies have suggested that gene therapy and immunotherapy also show good results in the treatment of glioma.

Metastasis is the main cause of death in cancer patients. It has been well known that the progression and metastasis of tumor cells are regulated by the activation of oncogenes and inhibition of anti-oncogenes. Many factors, such as vascular endothelial growth factor (VEGF)-mediated vascularization and epithelial–mesenchymal transition (EMT) is involved in the tumor metastasis. EMT mediates cell migration by decreasing cell adhesion, destructing the extracellular matrix, and reshaping the cytoskeleton. Small molecular inhibitors or monoclonal antibodies against VEGF have been used to inhibit the glioma growth, such as PTK/ZK (a VEGF receptor tyrosine kinase inhibitor), and Avastin (a monoclonal antibody against VEGF-A) [1,2]. RNA interference (RNAi) against the overexpressed genes in glioma also has the potential to suppress tumor progression. For example, Notch1 promotes the migration and invasion of glioma cells by activation of β-catenin and NF-κB signaling via AKT [3]. RNAi against Notch-1 can induce cell apoptosis and inhibit the proliferation of glioma cells [4].

Class III β-tubulin (β3-tubulin) is highly expressed in the central and peripheral nervous systems during the initial fetal and postnatal stages. In adult, β3-tubulin is mainly distributed in the neuron. Abnormal expression pattern of β3-tubulin may be associated with cancer [5]. Previous studies have found that β3-tubulin is involved in tumor aggressiveness. Hinsch and Quaas et al. found that overexpression of β3-tubulin is related to the aggressiveness of clear cell renal cell carcinoma and urinary bladder cancer [6,7]. Terry et al. found that, after androgen ablation, β3-tubulin expression increases in prostate cancer. This up-regulation of β3-tubulin is associated with the progression of prostate cancer to a castration-resistant state [8]. Hoflmayer et al. reported that β3-tubulin is overexpressed in upper gastrointestinal cancer types, and its expression is linked to tumor localization and prognosis in gastric cancer, tumor stage in the esophageal adenocarcinoma and the resection margin in esophageal squamous cell cancer [9]. Moreover, in breast cancer and ovarian cancer, high levels of β3-tubulin are related to low cell sensitivity to paclitaxel and microtubule stabilizer [10,11]. Consistent with this, in non-small cell lung cancer (NSCLC), low levels of β3-tubulin are associated with a better response rate, longer progression-free survival and overall survival rates [12]. At low concentrations, tubulin-binding agents can suppress overall microtubule dynamics in β3-tubulin knockdown cells, while at high concentrations, they induce cell apoptosis [13]. Unexpectedly, β3-tubulin knockdown has no significant influence on the microtubule dynamic instability [13]. Furthermore, in NSCLC, β3-tubulin can regulate cellular metabolism and stress response to promote cell survival and proliferation in the state of glucose starvation [14].

PRL-3 (regenerating liver-3 protein) phosphatase of, also known as PTP4A3, is a dual specificity phosphatase that belongs to a subfamily of protein tyrosine phosphatase (PTP). Three members (PRL-1, -2, and-3) of this subfamily constitute the novel small protein tyrosine phosphatases that share a highly homologous PTP motif, catalytic residues, and a unique modification of farnesylation. They can promote cell proliferation, migration, invasion, and metastasis. Particularly, many studies have uncovered the role of PRL-3 in cancer proliferation and metastasis in various tumors including colorectal, hepatocellular, prostate, breast, lung, ovarian, melanoma, and gastric cancers [15–22].

PRL-3 is involved in the proliferation and metastasis of tumor cells by a variety of mechanisms. Firstly, PRL-3 can promote EMT and facilitate the metastasis of tumor cells [23,24]. Besides, PRL-3 can affect cell motility by regulating the distribution of cytoskeleton and expression of integrin. Knockdown of PRL-3 leads to changes in the distribution of F-actin and α-tubulin cytoskeleton, and inhibits cell migration and invasion [25]. PRL-3 promotes migration and invasion of ovarian cancer cells by inhibiting the expression of c-Fos and integrin α2 [26]. A recent study has indicated that integrin β1 interacts with PRL-3 and is involved in PRL-3-induced migration [27]. Moreover, PRL-3 can regulate some essential cellular signalings and promote tumorigenicity. For instance, PRL-3 can induce the EGFR activation by downregulating the protein tyrosine phosphatase 1B (PTP1B) and promote cell growth, migration and tumorigenicity [28]. PRL-3 can dephosphorylate NHERF1 (Na+/H+ exchanger regulatory factor 1) and lead to an increase of NHERF1 and PTEN (a tumor suppressor with growth and survival regulatory functions) in the [25] cytoplasmic localization, leading to the malignant progression of melanoma [29]. Knowledge about the role of PRL-3 in glioma development is still limited. Previous studies have reported the expression of PRL-3 was up-regulated in glioma, mainly in grades III and IV. Meanwhile, matrix metalloproteinases [MMP2, MMP9, and membrane-type matrix metalloproteinase 1 (MT1-MMP)] were also detected in glioma tissues of grades IV and III [30]. This study proposed that PRL-3, MMP2 and MT1-MMP cooperatively promote glioma invasion [30].

Although the functions of PRL-3 in several tumors have been explored, its role in glioma tumorigenicity and the underlying molecular mechanism remained unclear. We hypothesized that the functional effects of PRL-3 on U87 cells may be related to the involvement of β3-tubulin, of which phosphorylation level could be regulated by PRL-3. Dephosphorylation of β3-tubulin facilitates assembly and reorganization of cytoskeleton, leading to cell migration and metastasis. In this study, we investigated the effects of PRL-3 overexpression and knockdown on the U87MG cell migration and invasion and explored the possible underlying mechanism.

## Methods

### Cells and culture conditions

293 T, U87MG, and U251MG glioma cell lines (Tianjin Medical Research Institute) were maintained in Dulbecco’s modified Eagle’s medium (DMEM) supplemented with 10% fetal bovine serum (FBS) and 1% penicillin/streptomycin. The cells were cultured at 37°C in a 5% CO_2_ incubator.

### Construction of PRL-3 overexpression and knockdown plasmids

To overexpress the PRL-3, a transient expression vector pIRES2-EGFP-PRL-3 was constructed. Briefly, cDNA was obtained from a total cellular RNA of 293T cells. The cDNA was amplified by PCR with primers: PRL-3-F1 (CGGAATTC**GCCACCA**TG GCTCGGATGAACCGCCC, *Eco*RI site is underlined; Kozak sequence is in bold) and PRL-3-R1 (CGGGATCCCTACATAA CGCAGCACCGGGTC, *Bam*HI site is underlined). The purified PCR products were ligated to pMD-18 T vector for sequencing. The PRL-3 fragments were then digested with *Eco*RI and *Bam*HI and cloned into the vector pIRES2-EGFP vector to generate pIRES2-EGFP-PRL-3.

To knockdown the PRL-3, a transient expression vector pGenesil-1-PRL-3 was constructed. PRL-3 shRNA was designed and synthesized based on the sequence (5’-CAAGACCCGGTGCTGCGTTAT-3’) obtained from GPP Portal Library (https://portals.broadinstitute.org/gpp/public/) with CTCGAG as loop. Sense strand: 5’-GATCCGCAAGACCCGGTGCTGCGTTATCTCGAGATAACGCAGCACCGGGTC TTGTTTTTTA-3’; anisense strand: 5’-AGCTTAAAAAACAAGACCCGGTGCTGCGTTATCTCGAGATAACGCAGCACCGGGTCTTGC-3’. shRNA expression plasmid pGenesil-1 containing EGFP and human U6 promoter was digested by restriction endonucleases *Bam*HI and *Hin*dIII. The oligonucleotide fragments of PRL-3 shRNA were annealed to form dsDNA and then ligated into linear pGenesil-1 to generate pGenesil-1-PRL-3. An irrelevant shRNA was used as a negative control. pGenesil-1-PRL-3 vector was then transfected into 293T cells to knockdown PRL-3.

### Lentivirus construction

To stably express or knockdown of PRL-3, lentiviral vectors were constructed. Briefly, PRL-3 shRNA (5’-CAAGACCCGGTGCTGCGTTAT-3’) lentiviruses, made by Gene Pharma (Shanghai, China) was used to knockdown PRL-3. The recombinant plasmid LV5-PRL3 was sequenced and transfected into 293T cells with packaging plasmids psPAX2 and pMD2.G. PRL-3 shRNA lentiviruses were used to knockdown of PRL-3. Similarly, a lentiviral vector overexpressing PRL-3 open reading frame (orf) was also produced. U87MG cells were infected with lentiviral particles at an MOI of 2 and selected using puromycin (3 μg/mL).

### Wound healing assay [31]

U87MG cells stably infected with PRL-3 overexpressing lentivirus or PRL-3 shRNA lentivirus were cultured in 6-well plates overnight. The cell monolayer was scratched using a pipette tip (200 ul) and allowed to culture for an additional 24 h. The cell migration distance was calculated using Image pro plus software (Media Cybernetics, Silver. Spring, USA).

### Matrigel invasion assay[32]

The upper chambers of wells were pre-coated with 200 μl of matrigel (Coning, 200 μg/ml). U87MG cells (2 × 10^4^) were plated in the upper chamber of each well. After culturing for 24 h, the cells that migrated to the lower chamber were fixed by methanol for 10 min and stained with 1% crystal violet for 15 min. After washing with PBS, the cells were observed using an inverted microscope.

### Expression and purification of PRL-3 recombinant protein

To express PRL-3, the plasmid pGEX-6P-1-PRL-3 was constructed. PRL-3 fragment was obtained by *Bam*HI and *Eco*R1 restriction enzyme digestion of the pcDNA3.1-PRL-3 plasmid (constructed previously in our lab). The PRL-3 fragment was cloned into the prokaryotic expression vector, pGEX-6P-1, to yield pGEX-6P-1-PRL-3, which was then transformed into BL21 cells and induced by IPTG (1 mM) for 2–3 h. Cells were washed by PBS and lysed by ultrasound for 2 min. The supernatants were incubated with ProteinIso GST resin according to the manufacture’s protocol (Transgene, Beijing, China). The purified proteins were boiled in a loading buffer for 5 min and separated by sodium dodecyl sulfate-polyacrylamide gel electrophoresis (SDS-PAGE) followed by staining using coomassie brilliant blue dye.

### Pull-down assay

U251MG cells (5 × 10^7^) were lysed using RIPA buffer containing 1% Triton X-100 and protease inhibitor cocktail on ice for 30 min. Prokaryotic expression plasmids pGEX-6P-1 and pGEX-6P-1-PRL-3 were transformed into BL21, and monoclones were selected and cultured overnight in LB medium containing Amp at 220 rpm in a shaking table. GST and recombinant protein GST-PRL-3 were obtained by transfer and IPTG induction for 2–3 hours, and bacterial precipitation was obtained by centrifugation in bacterial liquid. RIPA lysate containing lysozyme and PMSF was used to resuspend bacterial precipitation, then ultrasonic lysis was performed, centrifugation was performed, and the supernatant obtained was added with DTT and incubated overnight with Agarose beads at 4°C.The purified GST or PRL-3-GST proteins were incubated with the U251MG cell lysate overnight at 4°C. Agarose beads were washed for 5–8 times using RIPA buffer, resolved by SDS-PAGE and subjected to mass-spectrometric (MS) analysis. β3-tubulin was detected by Western blot.

### Immunoprecipitation assay

U251MG cells (5 × 10^7^) were lysed using RIPA buffer containing 1% Triton X-100 and cocktail on ice for 30 min. After centrifugation at 12,000 rpm for 10 min, U251MG cell lysate was obtained. Wash Protein A/G beads twice with PBS (PH7.4). Remove the supercleaner and mix 50% Protein A/G beads with PBS (PH 7.4). Add the cell lysate supernate to 200 μL Protein A/G beads in equal quantity, respectively. Centrifuge at 500 g at 4°C for 3 min. Transfer the supernate to A new centrifuge tube to remove Protein A/G beads.1 μg β3-tubulin primary antibody was added with PBS (PH7.4) to a total volume of about 500 μL. IgG with the same type of β-tubulin antibody was added in the control group as the control. Place on 3D rotary shaker and rotate overnight at 4°C low temperature. After overnight centrifugation at 500 g for 3 min, beads were collected and washed 3 times in pre-cooled PBS (PH7.4) with 800 μl added each time. Together with the positive control, the beads were boiled in a loading buffer and resolved by SDS-PAGE. PRL-3 was detected by Western blot.

### Western blot

Cells were lysed in a lysis buffer containing protease inhibitors for 30 min at 4°C. The samples were separated by 10% SDS-PAGE and transferred to PVDF membranes. The membranes were blocked with 5% skimmed milk in TBST solution [50 mM Tris, pH 8.0, 150 mM NaCl and 0.1% Tween-20 (v/v)] for 1 h at room temperature, and then probed by specific primary antibodies overnight at 4°C followed by anti-rabbit secondary antibody (1:5000; Beyotime) and anti‐mouse secondary antibodies (1:5000; Beyotime) for 1 hour at room temperature. The signals were detected by an enhanced chemiluminescence (ECL) reagent.

To ascertain whether PRL-3 can phosphoylate β3-tubulin, the silencing vector pGenesil-1-PRL-3 or overexpressing vector pIRES2-EGFP-PRL-3 was transfected into 293T cells. The cells were harvested at 48 h post-transfection (h p.t.) and lysed in RIPA buffer. Cell lysates were resolved using SDS-PAGE and then probed by Western blot using antibodies against PRL-3 (1:1000; Rabbit, Polyclonal, DF6747, Affinity), α-tubulin (1:2000; Rabbit, Polyclonal,112241AP, Proteintech), β3tubulin (1:1000; Rabbit, monoclonal, ab215037,abcam), p-S172-β3-tubulin (1:1000; rabbit, polyclonal, ab76286, abcam), and GAPDH (1:1000; mouse, Beyotime), respectively.

### Statistical analysis

Each experiment was repeated for three times. The data is expressed as mean ± SD. One-way ANOVA was performed to compare groups. P < 0.05 was considered statistically significant.

## Results

We hypothesized that PRL-3 enhances the migration and invasion of glioma cells by dephosphorylating β3-tubulin, and its dephosphorylation reorganize itself into cytoskeleton, leading to the cell migration. The purpose of the current study is to explore the effect of PRL-3 on the malignant degree of glioma cells and to explore its potential mechanism in cell migration. The study should provide an insight regarding the tumor treatment. First, we evaluated the effect of PRL-3 on invasion and migration of glioma cell line. Subsequently, based on mass spectrometry and bioinformatics, we identified β 3-tubulin as a downstream protein of PRL-3 that may be involved in cell migration. Finally, we confirmed that PRL-3 down-regulated the phosphorylation level of β3-tubulin and enhances the migration and invasion ability of glioma cells.

### Overexpression and knockdown of PRL-3 by lentiviruses in U87MG cells

**Figure 1.**
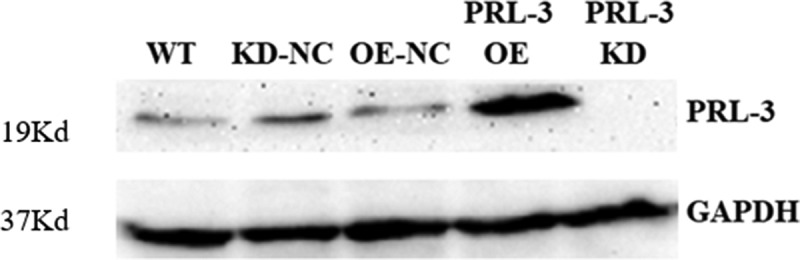
Western blot analysis of PRL-3 after overexpression and knockdown. The lentiviral vectors overexpressing PRL-3 (PRL-3 OE) or silencing PRL-3 (PRL-3 KD) were used to transduce the U87MG cells and the stably transfected cell lines were lysed for Western blot analysis. The empty vectors (OE-NC, KD-NC) were used as controls.

To determine the function of PRL-3 in glioma cell, lentiviral vectors were used to stably silence or overexpress the PRL-3. The expression levels of PRL-3 after overexpression or knockdown were assessed by Western blot. PRL-3 levels, upon transfection of the lentiviral-silencing vector expressing small hairpin RNAs (PRL-3 KD), were distinctly lower than upon transfection of the control vector (KD-NC) (Figure 1). Similarly, PRL-3 levels were markedly higher upon transfection with the overexpressing lentiviral vector (PRL-3 OE) than upon transfection with the control vector (OE-NC) (Figure 1).

### PRL-3 promotes migration of glioma cells

**Figure 2.**
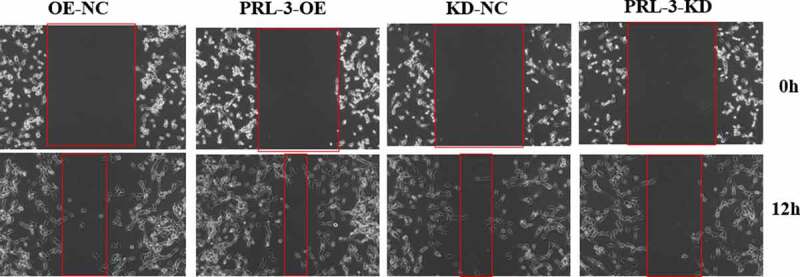
PRL-3 regulated the cell migration. The U87MG cell expressing PRL-3 or shRNA by lentiviruses were used for wound healing assay (a). Wound healing experiments showed the effect of overexpression and knockdown of PRL-3 on migration of U87MG cells.(b). Image Pro Plus software was used to analyze the migration distance.

Wound healing assay was performed to determine whether PRL-3 regulates the migration of glioma cells. Knockdown of PRL-3 significantly decreased the migration rates of glioma cells when compared to the control. Consistently, overexpression of PRL-3 led to a significant increase in the migration rates of glioma cells when compared to the control (Figure 2(a–b)).

### PRL-3 promotes invasion of glioma cells

**Figure 3.**
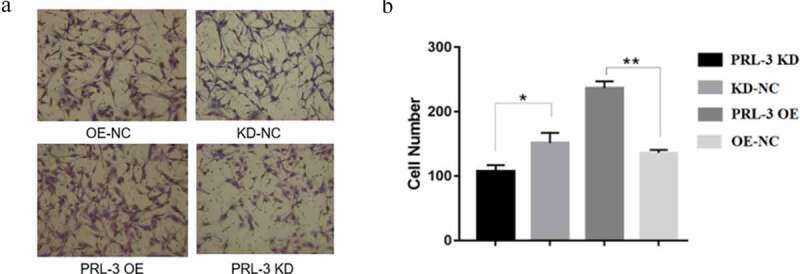
PRL-3 regulated the cell invasion. The U87MG cell transduced by lentiviruses expressing PRL-3 or shRNA were used for invasion assay (a-b). The upper chambers of 24-wells were pre-coated with matrigel, and then U87 cells (2ˣ10^4^) were plated in the upper chamber of each well. After 24 h, the cells migrated to the lower chamber were counted.

To determine whether PRL-3 can regulate the invasion of U87MG cells, a matrigel invasion assay was performed. There was a significant decrease in the number of the migrated cells after PRL-3 silencing (Figure 3(a–b)). On the other hand, PRL-3 overexpression caused a significant increase in the number of migrated cells (Figure 3(a–b)).

### Cloning, expression and purification of recombinant GST-PRL-3

**Figure 4.**
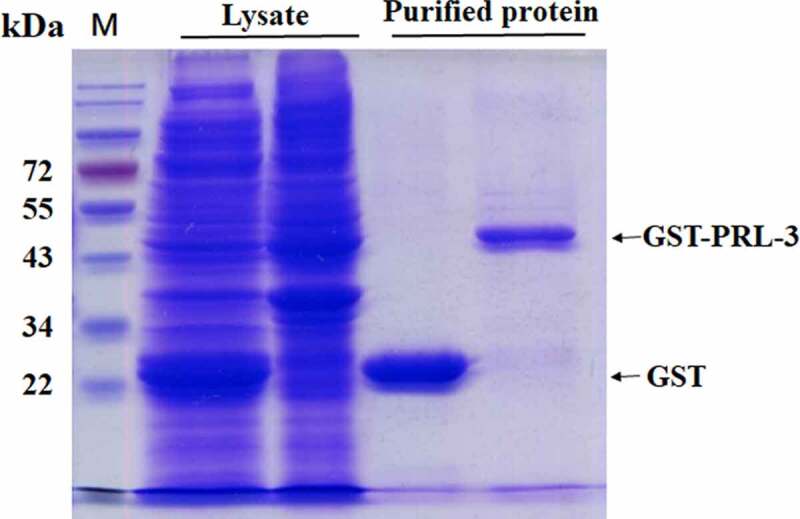
The expression of PRL-3 in BL21 cells. The PRL-3 gene was cloned into pGEX-6p-1 to generate pGEX-6p-1-PRL-3. pGEX-6p-1-PRL-3 or pGEX-6p-1 vectors were transformed to BL21 cells to express PRL-3-GST fusion protein or GST. The GST beads were used to isolate PRL-3-GST fusion or GST protein. The protein sizes were shown on the left.

PRL-3 protein was expressed using pGEX-6p-1 vector in *E. coli* BL21 cells. The recombinant fusion protein, PRL-3-GST, was successfully expressed in BL21 cells with a molecular weight of about 46 kDa (Figure 4). This fusion protein was then purified using GST agarose (Figure 4).

### Pull-down assay and validation of PRL-3 interaction proteins

**Figure 5.**
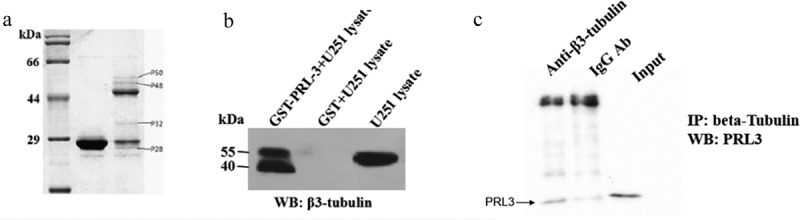
PRL-3 interacts with β3-tubulin. (a) Pull-down assay. PRL-3-GST fusion protein or GST protein was incubated with U251 cell lysates. After washing with lysis buffer, the proteins were resolved by SDS-PAGE and analyzed by mass-spectrometry. (b) Western blot analysis of β3-tubulin after performing pull-down assay. (c) IP assay. U251 cell lysate was immunoprecipitated by β3-tubulin antibody and the immunoprecipitated proteins were detected by anti-PRL-3 antibody.

A pull-down assay was performed in order to screen the proteins that interact with PRL-3 in U251MG cells. Several proteins were obtained in the SDS-PAGE gel after which the protein bands were further resolved and subjected to mass-spectrometric analysis (Figure 5(a)). β3-tubulin (p50 in Figure 5(a)) was confirmed by Western blot as one of the proteins that might interact with PRL-3 (Figure 5(b)).

To confirm the interaction of PRL-3 interact with β3-tubulin, an immunoprecipitation assay was performed. Figure 5(c) shows that PRL-3 was specifically immunoprecipitated by the β3-tubulin antibody. No protein band was detected for IgG antibody. This result indicated that PRL-3 physically interacts with β3-tubulin *in vitro*.

### PRL-3 regulates the phosphorylation level of β3-tubulin

**Figure 6.**
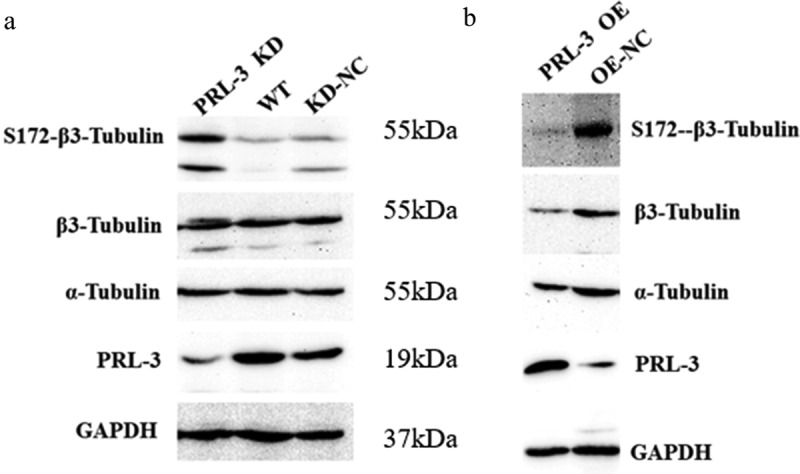
The detection of the phosphorylation level of β3-tubulin. The phosphorylation level of β3-tubulin was detected by Western blot. (a) Vectors expressing siRNA against PRL-3 or control SiRNA were transfected to 293T cells and the cell were harvested at 48 h post-transfection (h p.t.) The cell lysates were resolved by SDS-PAGE and then probed by antibodies against PRL-3, α-tubulin, β3-tubulin, p-S172-β3-tubulin, and GAPDH, respectively. (b) pIRES2-EGFP-PRL-3 or the control plasmids were transfected to 293T cells and the cells were harvested at 48 h p.t. The cell lysates were isolated by SDS-PAGE and then probed by the antibodies as (a).

To determine whether PRL-3 can phosphorylate β3-tubulin, Western blot was performed. Upon knockdown of PRL-3 by pGenesil-1-PRL-3, there was an increase in the phosphorylation level of β3-tubulin (Figure 6(a)). On the other hand, when PRL-3 was overexpressed by pIRES2-EGFP-PRL-3, there was a distinct reduction in the phosphorylation levels of β3-tubulin (Figure 6(b)). PRL-3 overexpression or knockdown had no distinct effect on the expression of α-tubulin. Unexpectedly, there was a decrease in the amount of total β3-tubulin upon PRL-3 overexpression. These results indicated that PRL-3 may regulate the expression and phosphorylation of β3-tubulin.

## Discussion

PRL-3 is a tyrosine phosphatase that is highly expressed in some tumors. PRL-3 has been found to be involved in the tumor metastasis. It is still unknown whether PRL-3 also plays a role in the carcinogenesis of glioma. In this study, we found that PRL-3 could promote the migration and invasion of glioma cells. It is found that PRL-3 interacted with and dephosphorylated β3-tubulin, which may be involved in cytoskeleton reorganization and facilitate cell motility. However, several questions still need to be resolved in the future. For instance, only an effective shRNA was used in the experiment, and only Hek293T cells were used instead of glioma cells to verify the results.

Previous studies have reported that PRL-3 is involved in tumorigenesis in a variety of tumor types. For instance, PRL-3 has been shown to promote growth and migration in human prostate cancer cells [17]. Moreover, PRL-3 is highly expressed not only in the prostate cancer tissue but the corresponding lymph node metastases as well, thus implying that it plays a role in the pathogenesis and malignity of prostate cancer [17]. Unn-Merete *et al*. reported that PRL-3 is overexpressed in human myeloma cells and plasma cells of patients. Silencing of PRL-3 reduced the cell migration, but had no detectable effect on the cell proliferation and cell cycle [33]. Consistent with these results, the present study also found that PRL-3 promoted glioma cell migration and invasion.

Of importance, PRL-3 can promote cell motility, invasion, and metastasis of some tumors, but the mechanism of PRL-3 mediating cell motility seems to be complicated to understand. Many proteins have been shown to be involved in the PRL-3 mediating metastasis. For example, Zhang *et al*. reported that PRL-3 regulated colorectal cancer progression by promoting AURKA ubiquitination and degradation through dephosphorylation of FZR1, leading to APC/C complex assembly [34]. Xing *et al*. suggested that Ubiquitin-specific protease 4 (USP4) stabilized PRL-3 by deubiquitination and is indispensible to potentiate the colorectal oncogenesis [35]. In colorectal cancer, PRL-3 has been shown to promote EMT by down-regulating the E-cadherin expression. PRL-3 mediates that by interacting with and down-regulating CDH22 expression, a member of the cadherin family [36]. In addition, KCNN4 plays a role in the PRL-3-mediated EMT in colorectal cancer. KCNN4 not only decreases E-cadherin but also increases snail expression via an increase in the intracellular calcium levels and activation of GSK-3β signaling [24]. Peng et al. reported that PRL-3 controlled cell motility, invasion and metastasis in colon cancer through integrin β1-ERK1/2-MMP2 signaling [37]. Integrin β1 knockdown inhibited the activity of ERK1/2 and suppressed the PRL-3-induced motility and invasion [37].

The present study indicated that PRL-3 interacts with and dephosphorylates β3-tubulin at Ser 172. Previously, it is unknown whether the interaction of PRL-3 with β3-tubulin is involved in the migration and invasion of glioma cells, and only a few studies have revealed the roles of β3-tubulin in the tumor progression. For instance, Hinsch and Quaas *et al*. reported that overexpression of β3-tubulin is related to aggressiveness of clear cell renal cell carcinoma and urinary bladder cancer [6,7]. Other studies, including knockdown and overexpression of β3-tubulin, will be performed to elucidate the role of β3-tubulin in PRL-3 mediating cell migration and invasion. Meanwhile, whether PRL-3 phosphatase activity plays an important role in cell migration also needs to be explored in the future.

The current study also indicated that PRL-3 appeared to regulate the phosphorylation levels of β3-tubulin at Ser 172. The function of β3-tubulin phosphorylation in the glioma progression is still elusive. Previous study suggested that the increased expression of PRL-3 can promote the migration and invasion of endometrial stromal cells by reorganizing the F-actin and α-tubulin cytoskeleton [25]. Further studies are needed to explore the mechanism by which PRL-3 could possibly regulate the phosphorylation of β3-tubulin and the cytoskeleton changes that may be involved in PRL-3-mediated cell migration.

PRL-3 is involved in the migration and invasion of glioma cells, and β3-tubulin may play an important role in PRL-3-mediated carcinogenesis of glioma. Further studies are also needed to determine whether PRL-3 is also implicated in the metastasis of glioma *in vivo*. A deep understanding of the mechanism of glioma development will provide novel clues and methods for glioma treatment.

## Conclusion

In conclusion, PRL-3 enhances the malignancy of glioma cells, in which dephosphorylation of β3-tubulin by PRL-3 may play a critical role in cell migration.

## HIGHLIGHTS

- PRL-3 can bind to β3-tubulin in vitro and in vivo.
- PRL-3 may downregulate phosphorylation of serine 172 in β3-tubulin.
- PRL-3 can enhance the migration and invasion of glioma cells.
